# Ganga hospital open injury severity score as a predictor of early failure of limb salvage among gustilo type III A and B tibia fractures in Uganda: a prospective cohort study

**DOI:** 10.1186/s12893-025-02811-1

**Published:** 2025-02-21

**Authors:** Maxwel Dancan Okuku, Umaru Kabuye, Stephen Angira Khadolwa, Abubakar Mohamed Aweis, Okedi Francis Xaviour, Demoz Abraha, Charles Quealee, Anthony Ayotunde Olasinde, Ibe Michael Usman

**Affiliations:** 1 Department of Surgery, Kampala International University Western Campus, Bushenyi, Uganda; 2 Department of Orthopaedic Surgery, Federal Medical Centre, Owo, Ondo State Nigeria; 3 Department of Human Anatomy, Kampala International University Western Campus, Bushenyi, Uganda; 4 Department of Nursing, Mountains of the Moon University, Fort Portal, Uganda

**Keywords:** Gustilo type III tibia fractures, GHOISS, Limb-salvage surgery

## Abstract

**Background:**

Despite advances in antibiotic therapy and microsurgery, the management of Gustilo and Anderson type IIIA and B open tibia fractures continues to pose a considerable challenge in developing countries. This has evolved from historical immediate amputation to modern approaches that prioritize both aesthetics and functional outcomes. Nonetheless, a consensus on limb salvage versus amputation remains elusive, prompting the development of prognostic limb scoring systems. Our study assessed the predictive accuracy of the Ganga Hospital Open Injury Severity Score (GHOISS) for early failure of limb salvage in Gustilo type IIIA and B tibia injuries.

**Methods:**

This was a prospective study that examined open tibia fractures at two tertiary hospitals in the emergency and orthopaedic units between June and October 2023. Fifty-three (26 IIIB and 27 IIIA) satisfied the study inclusion and exclusion criteria. Four injuries (type IIIA) in paediatric patients that had met the inclusion criteria were excluded from analysis to ensure homogeneity and generalizability of the results due to their small numbers. The severity of injury for each Gustilo type III A and B tibia fracture was determined using the GHOISS, and limb salvage decisions were made irrespective of the GHOISS. Follow-up was extended for up to fourteen days to assess the necessity of secondary amputation in salvaged limbs.

**Results:**

Among 49 Gustilo type IIIA and B tibia fractures, 43 were successfully salvaged, while 6 necessitated amputation (4 primary, 2 secondary). A GHOISS of 13 demonstrated maximum specificity (90.7%) and sensitivity (83.3%) in predicting amputation, with an AUC of 0.923 (95% CI 0.804–0.977), indicating strong discriminatory accuracy.

**Conclusion:**

The GHOISS reliably predicted outcomes in patients with Gustilo type IIIA and B tibia fractures, with a score of 13 demonstrating optimal sensitivity and specificity above which early failure of limb salvage is anticipated.

## Background

Gustilo type III tibia fractures pose substantial socioeconomic challenges, particularly in resource-limited nations [2]. The nationwide prevalence in German has been estimated at 13.8% [1], The incidence of these fractures is projected to increase due to factors such as increased automobile use and population growth [3]. Consequently, these injuries inflict significant morbidity, greatly impacting the quality of life for those affected [4, 5].

Historically, the management of Gustilo type III tibia fractures necessitated an emergency amputation prior to the era of safe surgical techniques and antimicrobial agents [2, 6]. Advancements in antibiotics, aseptic techniques, and microsurgical methods have transformed the approach used for treating these fractures, emphasizing cosmetic and functional restoration [7]. Despite this paradigm shift, a consensus has yet to be reached regarding limb salvage versus amputation [2]. Erroneous clinical decisions can result in unnecessary or delayed amputations which carry substantial morbidity and mortality risks [2, 8]. Thus, there is a pressing need to develop a predictive scoring system capable of accurately predicting the need for amputation or limb salvage [3].

Several prognostic limb scoring systems, such as the Gustilo-Anderson classification system (1976), MESS (1990), and GHOISS (2006), have been introduced over the years [9–11]. Currently, the MESS is the most widely adopted system [8]. However, MESS exhibits suboptimal performance in patients without vascular injuries, specifically Gustilo III A and III B fractures. To address this limitation, Rajasekaran et al. developed the GHOISS in 2006, primarily for Gustilo type III A and III B tibia fractures [12]. GHOISS has demonstrated superior clinical decision-making capabilities in more developed regions [12]. However, its effectiveness in resource constrained settings, such as Africa, where Uganda is located, remains uncertain due to limited access to advances in microsurgical techniques and a shortage of specialized surgeons. The availability of vascular and plastic surgeons who play a vital role in the management of these complex injuries is a key factor in these areas. Uganda has a high prevalence of Gustilo type III tibia fractures (13.7%), a figure expected to rise with increasing industrialization and use of road traffic [13]. Therefore, our study aimed to assess the discriminatory accuracy of the GHOISS at predicting early failure of limb salvage in Gustilo type III A and B tibia fractures in Uganda.

## Methodology

This was a prospective study that examined open tibia fractures at two tertiary hospitals in the emergency and orthopaedic units between June and October 2023 using Ganga Hospital Open Injury Severity Score (GHOISS). We included Gustilo class III tibia fractures who presented within 24 h of injury irrespective of the age or fracture location and excluded those who present after having initial debridement or surgery of the fracture at another hospital, traumatic amputations as well as those with significant injury to large vessels that would repair to re-establish perfusion to the distal part of the affected leg.

A power analysis was conducted to determine the required sample size, considering the limited population of 54 patients available for evaluation. Our analysis was informed by previous research on the Ganga Hospital Open Injury Severity Score (GHOISS), aiming to replicate the reported sensitivity of 75% and specificity of 93.75% in predicting amputation versus limb salvage in Gustilo type III A and B tibia fractures. Assuming a precision of ± 5% and 80% power, we initially calculated that a sample size of 288 fractures would be needed to estimate sensitivity and 90 fractures to estimate specificity. However, due to the finite population size of 54 fractures, we applied a correction to adjust the sample size, reducing it to 47 fractures for sensitivity and 34 fractures for specificity. Therefore, we based our study on a final adjusted sample size of 47 fractures to ensure sufficient power to estimate both sensitivity and specificity with adequate precision resulting into 52 fractures after considerations of the 10% loss to follow up.

During this period, a total of 87 patients with 88 open tibia fractures presented at emergency and orthopaedic units of the two hospitals. These were according to Gustilo and Anderson Classification system classified as follows. Six were Gustilo and Anderson class I, 22 were class II, 30 were class IIIA while 27 and 3 were class IIIB and IIIC respectively. One patient had bilateral type IIIB tibia fractures. Type I, II and IIIC fractures were excluded leaving 57 IIIA and IIIB fractures.

Of the 57, 4 (3 class IIIA and 1 class IIIB) were excluded because 2 presented after 24 h from injury time and the other 2 presented after debridement from peripheral facilities. To ensure homogeneity and generalizability of the results, 4 cases in paediatric patients were further excluded due to their small numbers leaving 49 fractures for analysis. Participants and/or their legal representatives signed an informed consent form prior to recruitment into the study. This study was approved by the Mulago Hospital Research and Ethics Committee (MHREC 2537). Participants were evaluated and managed according to the standard management protocols for trauma patients at each of the institutions. Temporary stabilization of the fractures using locally fabricated splints and crepe bandages was performed upon arrival at the emergency departments of the two tertiary hospitals. Patients with injured limbs which after fluid resuscitation still had unappreciable posterior tibialis and dorsalis pedis arterial pulses on physical exam underwent a doppler ultrasound scan to rule out presence of vascular injury requiring repair. Further procedures were performed upon transfer to the orthopedic departments as quickly as possible. Immediate wound debridement was performed with amputation or salvage according to the discretion of the operating team. The GHOISS scoring was performed preoperatively and prospectively by a trained general surgery resident at the study centers who was blinded to the clinical management plan. The decision to amputate or salvage was made by a team of orthopaedic and general surgeons, considering injury severity and the patient’s overall condition. The facilities prioritize limb salvage whenever feasible; however, amputation is performed when the risk of complications or mortality is substantial. Skeletal stabilization was achieved by long leg casting with a window over the wound, external fixation or ORIF with interlocking nails for those who were salvaged depending on the anatomy of the fracture.

Patients received tetanus prophylaxis and a 3-day course of intravenous antibiotics (ceftriaxone and a short course of gentamicin) upon arrival. The antibiotics were continued if the wound demonstrated features of infection. A uniform analgesia protocol consisting of intravenous paracetamol and pentazocine was initiated perioperatively and continued into the postoperative period. Wound care and other necessary treatments for associated injuries were also provided as needed. Patients whose limbs were salvaged were followed up for 14 days to establish whether they underwent secondary amputation, as most secondary amputations were reported to occur within this time [14]. Early failure of limb salvage was defined by either primary or secondary amputation.

Participant characteristics, such as age, sex, medical factors (i.e., admission systolic blood pressure, DM or cardio-respiratory disease), and injury details, such as GHOISS scores and treatment outcomes, e.g., amputation or salvage, were entered into the proforma designed for the study.

The GHOISS score considers the extent of damage to different limb elements (skin, bones, and muscles), assigning a score of 1 to 5 for each [3]. Additional points (2 each) are added for systemic factors affecting patient outcomes. The total score is calculated by summing these values (Table 1) [3, 8]. This total score categorizes open injuries into four groups: Group I [3–5], Group II [6–10], Group III [11–15], and Group IV (16–29) [3, 8].

**Table 1:** The ganga hospital open injury severity score

Category	Description	Score
**Covering Structures**: Skin and Fascia		
Wound with no skin loss and not over the fracture site	No skin loss; not over the fracture site.	1
Wound with no skin loss and over the fracture site	No skin loss; over the fracture site.	2
Wound with skin loss and not over the fracture site	Skin loss; not over the fracture site.	3
Wound with skin loss and over the fracture site	Skin loss; over the fracture site.	4
**Functional Tissues**: Musculotendinous and Nerve Units		
Partial injury to Musculotendinous	Partial injury to musculotendinous unit.	1
Complete but repairable Musculotendinous injury	Complete but repairable injury to musculotendinous unit.	2
Irreparable Musculotendinous injury or complete injury to posterior tibial nerve	Irreparable musculotendinous injury, partial loss of compartment, or complete injury to posterior tibial nerve.	3
Loss of one compartment of the Musculotendinous unit	Loss of one compartment of the musculotendinous unit.	4
Loss of two or more compartments or subtotal amputation	Loss of two or more compartments, or subtotal amputation.	5
**Skeletal Structures**: Bones and Joints		
Transverse/oblique fracture or butterfly fragment < 50% circumference	Transverse/oblique fracture or butterfly fragment less than 50% of circumference.	1
Large butterfly fragment > 50% circumference	Large butterfly fragment greater than 50% of circumference.	2
Comminuted or segmental fracture without bone loss	Comminuted or segmental fracture with no bone loss.	3
Bone loss < 4 cm	Bone loss of less than 4 cm.	4
Bone loss > 4 cm	Bone loss of more than 4 cm.	5
**Co-morbidities**: Add 2 points for each condition present		
Injury to debridement time interval greater than 12 h	Injury with a debridement time interval greater than 12 h.	2
Sewage or organic contamination or farmyard injuries	Injuries involving sewage or organic contamination, or farmyard injuries.	2
Age greater than 65 years	Age of the patient greater than 65 years.	2
Drug-dependent diabetes mellitus or cardio-respiratory disease leading to increased anesthetic risk	Drug dependency, diabetes mellitus, or cardio-respiratory disease increasing anesthetic risk.	2
Polytrauma involving chest or abdomen with injury severity score > 25 or fat embolism	Polytrauma involving the chest or abdomen with an injury severity score greater than 25 or fat embolism.	2
Hypotension with systolic blood pressure < 90 mmHg at presentation	Hypotension with systolic blood pressure less than 90 mmHg at presentation.	2
Another injury to the same limb or compartment syndrome	Another injury to the same limb or compartment syndrome.	2
**Decision Guidelines**		
Limb salvage is advisable for a score ≤ 14		N/A
Amputation is advisable for a score ≥ 17		N/A
Scores of 15 and 16 fall into a “gray zone” where decision depends on patient-specific factors		N/A

The sensitivity and specificity of the GHOISS and treatment outcome (amputation vs. salvage) were the primary and secondary outcomes, respectively. Primary amputation was considered if performed at the initial debridement, and secondary amputation was considered if performed at any time after the initial debridement but not after 14 days.

The data were entered into and summarized in Microsoft Excel and subsequently transferred to STATA version 14.2 for analysis. The means and standard deviations were used if the variable was normally distributed or the medians if the variable was skewed for continuous variables. Percentages and frequencies were used for categorical variables. Tables and figures where appropriate were used to present the results. To determine the ability of the GHOISS to distinguish patients for amputation, area under the receiver operating characteristic (ROC) curves were generated. The score estimate on the ROC curve whose sensitivity and specificity had the maximal Youden’s index ([Sensitivity + specificity] – 1) was considered to be the optimal threshold score, and its corresponding sensitivity, specificity, and area under curve (AUC) were reported.

## Results

The study included 48 adult patients; one had bilateral Gustilo type III tibia fractures making a total of 49 fractures for analysis. The male to female ratio was 5:1:. The mean age was 37 years, with a standard deviation (SD) of 12.9 (95% CI 33.309–40.732 ). The patient age ranged from 20 to71 years. Twenty-one (43% ) of the fractures, patients had no associated co-morbid factors, while in 28 (57% ) of the fractures, patients had one or more co-morbid factors according to GHOISS system. The median, mean, standard deviation (SD) and confidence intervals (CIs) of the GHOISS score, bone score, musculotendinous core and skin score are shown in Table 2.

**Table 2:** The means, standard deviations and medians of GHOISS, bone, skin and musculotendinous injury scores

Variable	Mean	Median	SD	[95% Conf. Interval]
GHOISS	9.571	9.000	3.291	8.626–10.517
Bone score	2.408	2.000	0.762	2.189–2.627
Musculo-tendinous score	2.061	2.000	0.517	1.913–2.210
Skin score	2.857	3.000	0.791	2.630–3.084

**Fig. 1:**
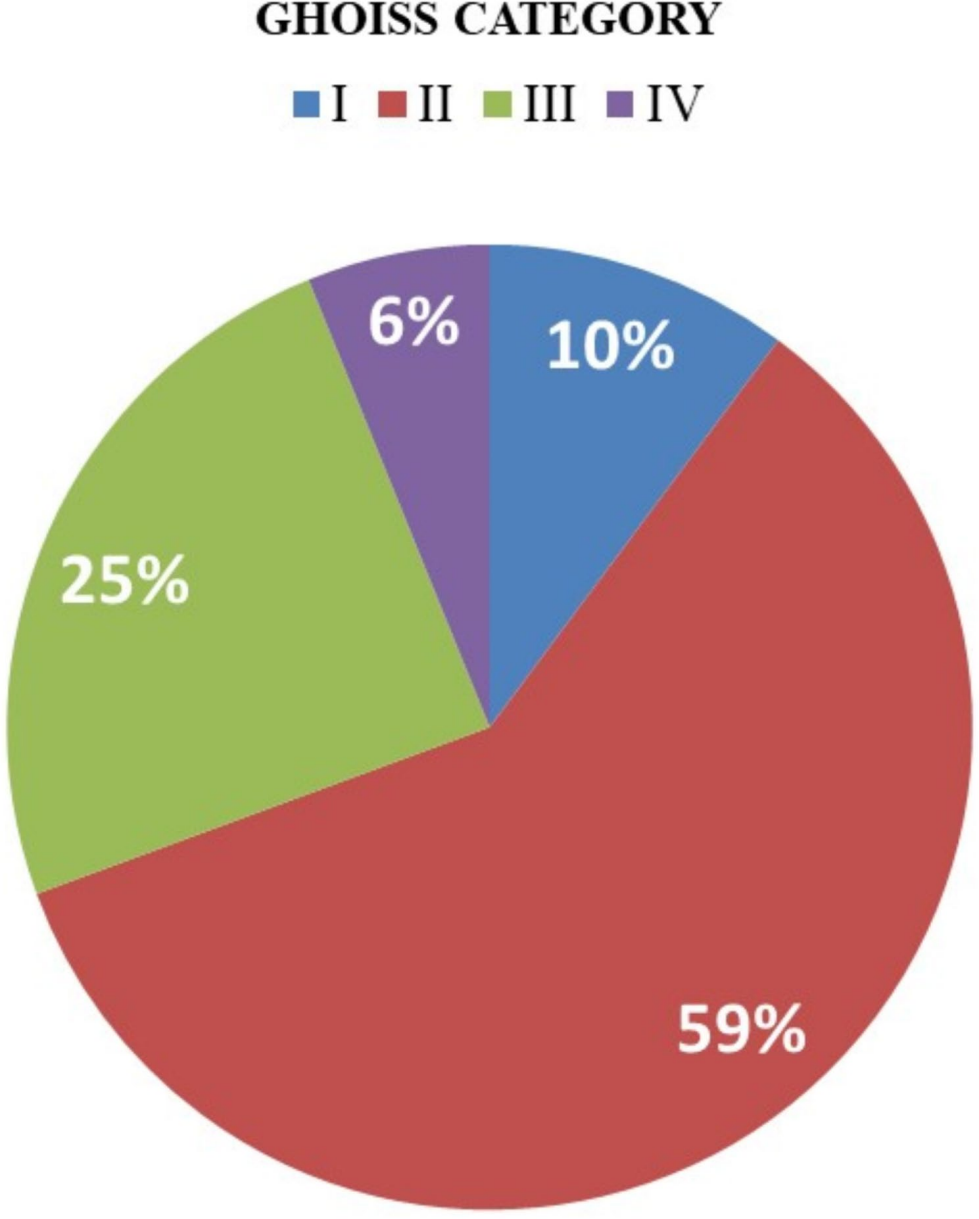
Distribution of fractures according to GHOISS category

Most injuries (29/49; 59%) were categorized as Group II in the GHOISS classification, while Group IV injuries were the least common (3; 6%) as shown in Fig. 1. Early successful limb salvage was achieved in 43 (88%) patients, while 6 (12%) required amputation. Four primary amputations had GHOISS scores of 10, 13, 17, and 19. Two secondary amputations had scores of 10 and 13, performed due to severe soft tissue infections. The analysis of limb amputations based on GHOISS scores revealed distinct patterns. Primary amputations were associated with GHOISS scores of 10, 13, 17, and 19. In contrast, secondary amputations had GHOISS scores of 10 and 13. The mean GHOISS score for amputation was 14.8 (95% CI 11.295–18.371).

**Fig. 2:**
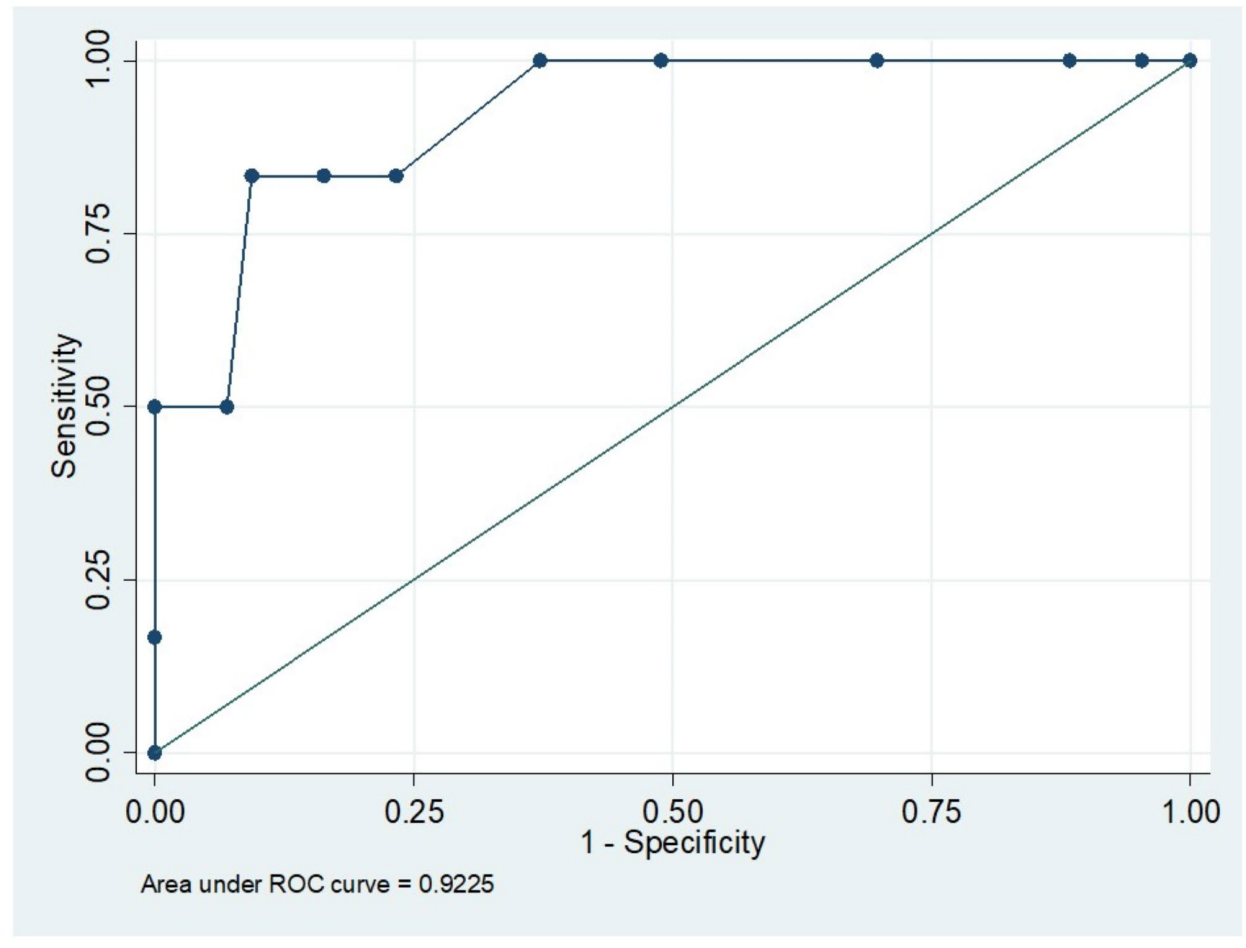
ROC curve (blue line) of GHOISS score vs. amputation

**Fig. 3:**
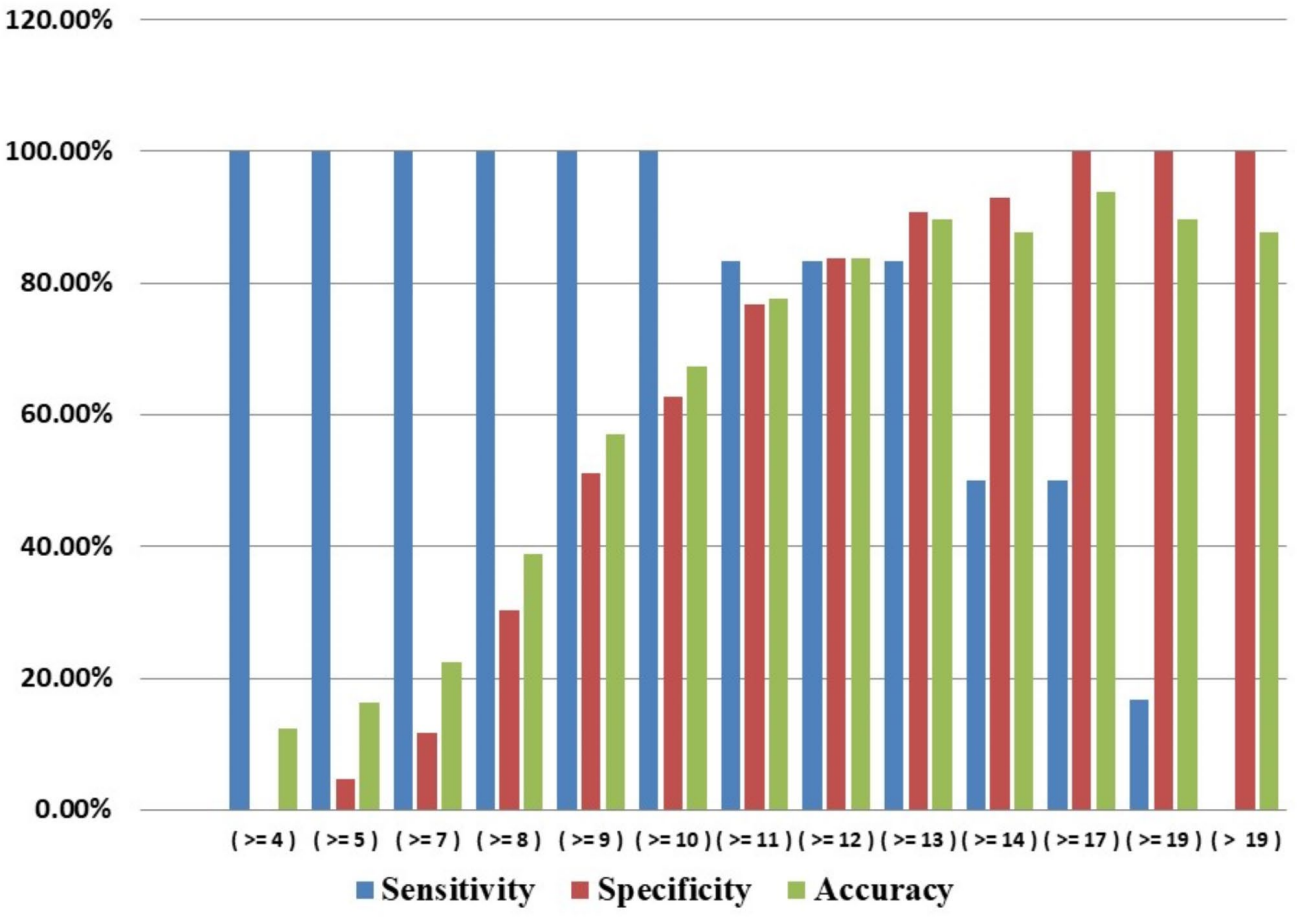
Bar chart showing the accuracy of the GHOISS in predicting amputation at different cut offs

**Fig. 4:**
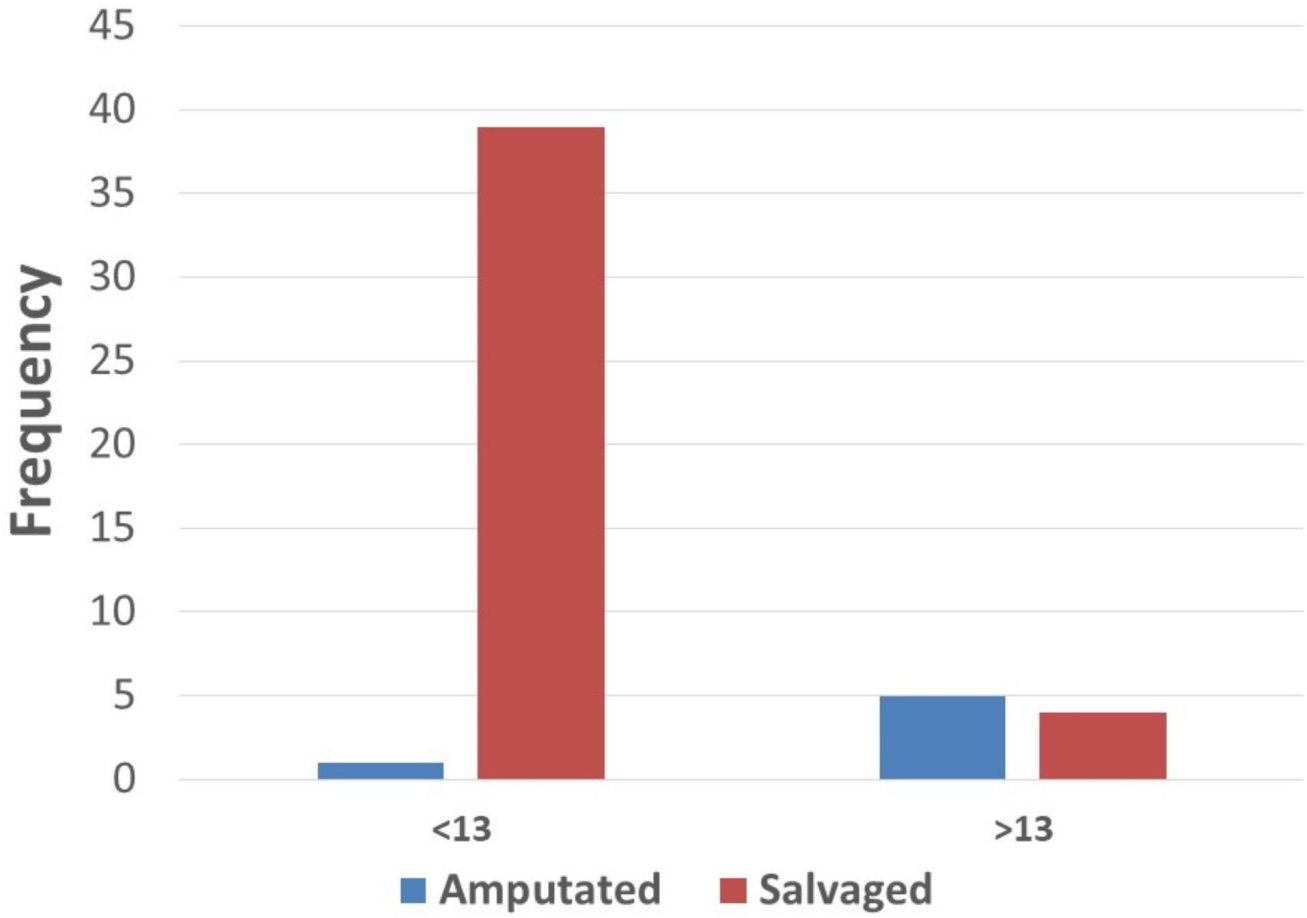
GHOISS score versus amputation

Binary logistic regression analysis demonstrated that the GHOISS score was a significant predictor of amputation (*p* < 0.001).The GHOISS score demonstrated strong predictive ability for amputation, with an area under the curve (AUC), sensitivity and specificity of 0.923 (95% CI 0.804–0.977), 83.3% and 90.7%, respectively, at a threshold score of 13, as shown in Figs. 2 and 3. Thirty-nine of 40 (97.5%) fractured limbs with GHOISS scores less than 13 were salvaged; however, only 5/9 (55.6%) of the limbs with GHOISS scores 13 and above underwent amputation, as shown in Fig. 4. The mean GHOISS for successful salvage and amputation were 8.8 (SD = 2.6, 95% CI 8.051–9.623) and 14.8 (SD = 3.4, 95% CI 11.295–18.371) respectively.

## Discussion

This study presents the first application of the Ganga Hospital Open Injury Severity Score (GHOISS) in Uganda and Africa to prognosticate the need for amputation in Gustilo type III A and B tibia fractures. This is significant because, until now, no validated scoring system has been widely utilized in the region to guide limb salvage decisions in severe open tibia fractures. The absence of a standardized approach has likely contributed to inconsistent clinical decisions, delayed amputations, and increased morbidity. By introducing GHOISS, we provide a structured, evidence-based tool that can improve early decision-making, optimize resource allocation, and potentially reduce the socioeconomic burden of prolonged hospital stays and failed salvage attempts. The management of Gustilo type III tibia injuries has long been a critical aspect, often leading to challenging dilemmas of limb salvage or amputations decisions. Incorrect decisions, such as unnecessary or delayed amputations, have been associated with significant morbidity and mortality, as documented in previous studies [4, 5, 15, 16]. Unsuccessful limb salvage can result in prolonged suffering, including pain, extended hospitalizations, increased surgical interventions, and financial strain [11].

The GHOISS, developed in 1994 and validated in 2006 by Rajasekaran and colleagues, was designed to address the challenge of limb salvage versus amputation in Gustilo type III patients [2, 17, 18]. Previous studies have demonstrated the effectiveness of the GHOISS in predicting the need for amputation or limb preservation in Gustilo type IIIB and IIIA injuries [12]. Furthermore, the GHOISS has shown equal specificity and sensitivity for limb salvage or amputation in both adults and children [8, 18]. Specifically, a GHOISS of 17 or higher has been associated with the highest sensitivity and specificity for limb loss, while a score of 14 or lower has been predictive of successful salvage [2, 18].

The distribution of injuries according to the GHOISS classification system in our study aligns with previous literature, particularly in the predominance of Group II injuries [3, 15, 19]. Notably, our findings are consistent with the prospectively designed studies, which also reported Group II injuries as the most common. However, there is a single retrospective study that diverges from this pattern, indicating Group III injuries as predominant in its cohort [8].

In terms of demographics, our study, along with previous research, underscores that males are more frequently affected by these injuries compared to females. This observation is consistent across various studies and can be attributed to the higher-risk activities and lifestyles of men in the society. Moreover, the presence of one or more co-morbidities, as assessed by the Ganga score, is notable in a significant number of patients, as evidenced by previous studies. This highlights the importance of considering co-morbid conditions in the management and prognosis of these injuries. The observed salvage rate of 88% in our study of Gustilo Type III tibia injuries are consistent with previous studies by Basu et al. (2022), Madhuchandra et al. (2015), Rajasekaran et al. (2006), and Venkatadass et al. (2017), which reported successful salvage rates exceeding 86%. These studies also observed a strong association between GHOISS category and outcome, with most amputations occurring in patients classified in group III or IV, with GHOISS scores exceeding 14. Notably, Madhuchandra et al. (2015) reported an exceptionally high salvage rate of 97.5%, attributed to a cohort dominated by patients with mild injuries, with only one limb having a GHOISS score greater than 15. Secondary amputations in our study, as in others, were primarily attributed to persistent infections. However, our study included 6 amputations out of 49 cases, which may have led to wider confidence intervals, thereby reducing the precision and statistical power of our sensitivity and specificity estimates.

Specifically, a GHOISS score of 17 or higher was found to have the highest sensitivity and specificity for limb loss, while a score of 14 or lower predicted successful salvage. Notably, a “gray zone” with scores between 15 and 16 was identified, where the decision depends on the surgeon’s expertise and the patient’s financial capacity to cover reconstruction costs [2].

Similar to earlier studies like that by Elniel and Giannoudis (2018), our study revealed a significant association between GHOISS and the likelihood of amputation. Specifically, we identified a GHOISS score of 13 as the threshold above which amputation should be considered in our setting. Interestingly, we observed one case where a limb with a GHOISS score of 10 underwent secondary amputation despite initially predicting salvage.

Our threshold score of 13 is slightly lower than 14 reported in previous studies. This difference may be attributed to variations in healthcare settings and treatment approaches. Previous studies often involved highly specialized centres employing orthoplastic approaches, where orthopaedic and plastic surgeons collaborated closely throughout the treatment process, from initial debridement to reconstruction different from the approach used in our setting with no involvement of plastic surgeons due to their scarcity. Importantly, our study did not identify a gray zone. Since previous studies that reported a gray zone had larger sample sizes, we attribute its absence in our study to the smaller sample size [2, 18]. The sample size used for the present study constitute a limitation which can potentially increase possibility of a type II error, hence conducting a similar study with a larger sample size will not be out of place. The short follow up period due to duration of the study also constitute a limitation to the study, since we are not able capture amputation that may have been after an extended period of time; we therefore recommend future studies with larger sample sizes and longer follow ups. Despite the above-mentioned limitations, the present study used a prospective design and validated tool; hence, laying a good foundation for further study on Gustilo Type III A and B Tibia Fractures from this part of the world with limited documented cases.

## Conclusion

The GHOISS score proved to be a reliable predictor of outcomes for Gustilo type III tibia fractures; therefore, it should be integrated in decision-making and prognosticating the treatment outcomes for these injuries. Notably, a GHOISS score of 13 exhibited the highest sensitivity and specificity above which early failure of limb salvage is anticipated in this setting.

## Data Availability

The datasets analysed during the current study are available from the corresponding author upon reasonable request.

## Abbreviations

AUC: Area under the curve
Dr: Doctor
GHOISS: Ganga Hospital Open Injury Severity Score
JRRH: Jinja Regional Referral Hospital
KIUTH: Kampala international University teaching and Research Hospital
MESS: Mangled extremity severity score
OTF: Open tibia fracture
ROC: Receiver operator curve
SD: Standard deviation
